# Unravelling the tapestry: Cross-cultural insights into intelligence and creativity

**DOI:** 10.1371/journal.pone.0320942

**Published:** 2025-05-06

**Authors:** Vlada Alekseevna Repeykova, Anatoliy Vladimirovich Kharkhurin, Sergey Rostislavovich Yagolkovskiy

**Affiliations:** Department of Psychology, HSE University. Moscow, Russia; RAK Medical and Health Sciences University: Ras Al Khaimah Medical and Health Sciences University, UNITED ARAB EMIRATES

## Abstract

Literature suggests multiple relationships between creativity and intelligence, each supported and contradicted by empirical evidence. The current study investigated cultural variations in the relationship between divergent thinking (DT) and fluid intelligence (Gf). Study participants were students from the most reputable universities in Russia (*N* = 53) and the United Arab Emirates (UAE; *N* = 53). The DT test measured their creative potential, the Abbreviated Torrance Test for Adults (ATTA), from which five indicators were extracted for further analyses: composite Creativity Index (CI), fluency, originality, elaboration, and flexibility. The Culture Fair Intelligence Test (CFIT) assessed participants’ Gf. We found a significant negative relationship between Gf score and CI, fluency, elaboration, and flexibility. The results suggest a non-linear trend, which was addressed accordingly. Adding a cultural component into the model explained substantial variance in DT and Gf scores. The Russian sample outperformed the UAE sample on all DT components, while the UAE sample outperformed the Russian sample on the Gf. Samples were different, predominantly on the fluency component, and were more similar in originality. Overall, results suggest the complex interplay between DT and Gf in cross-cultural settings.

## Introduction

Creativity and intelligence, as fundamental aspects of human cognitive capabilities, have long been subjects of interest in psychological research [1]. The existing literature reveals a myriad of relationships between different components of creativity (e.g., divergent thinking) and intelligence (e.g., fluid intelligence), ranging from correlational to causal and varying in strength [2]. Moreover, some studies report linear associations, while some suggest more complex, non-linear patterns [3]. The complexity extends further when various contextual factors are taken into account. One such salient factor is culture, as cultural norms, values, and societal expectations around creativity and intelligence might influence how these constructs are perceived and developed, shaping their relationship [4]. This research shows how the interplay between creativity and intelligence may display unique dynamics specific to Russian and United Arab Emirates (UAE) cultural contexts.

### Relationship between divergent thinking and fluid intelligence

Creativity and intelligence are multifaceted constructs with interrelated components [5]. Creativity is often linked to generating original, useful ideas and involves divergent thinking (DT), which includes fluency (producing many solutions quickly), originality (creating unique ideas), elaboration (developing ideas in detail), and flexibility (adopting various approaches) [6]. Intelligence is generally defined as the capacity for problem-solving and learning, encompassing fluid intelligence (Gf), the ability to solve novel problems through reasoning [7].

Some research considers intelligence a key component of creativity, emphasizing the role of fluid reasoning in creative cognition [8,9]. A reciprocal relationship suggests creativity may be a sub-facet of intelligence [10,11]. The “indifference of the indicator” phenomenon proposes that all cognitive functions serve as intelligence tasks, with scores from various assessments contributing to intelligence [12]. Traditionally, the creativity-intelligence link is examined in terms of strength, direction, and linearity.

A pivotal meta-analysis found a correlation coefficient of.17 between DT and Gf [13]. While early studies suggested correlations between DT and Gf ranged from negligible to small [14,15], later research indicated modest to substantial correlations [12,16–19].

The variability in correlation coefficients may stem from unknown biases and differences in how correlations are assessed. For example, stronger relationships may be observed when DT and Gf are evaluated at the same level (e.g., general intelligence vs. general creativity) rather than at different cognitive dimensions [20]. Additionally, past research has primarily focused on verbal DT tasks, showing weaker correlations with figural tasks [21]. The relationship’s strength can also vary based on whether DT is assessed with quality-focused or quantity-focused instructions [22].

While the relationship size varies, the direction is generally positive [6,12,20,23]. The nature of the relationship – linear or non-linear – has also been debated. The Necessary But Not Sufficient Hypothesis suggests that high creativity is rare among those with low intelligence, indicating a weaker correlation at higher intelligence levels, resulting in a triangular scatterplot pattern [17]. Previous analytical procedures used to test the Necessary But Not Sufficient Hypothesis were criticized [24]. The Threshold Hypothesis posits a positive correlation between creativity and intelligence only up to a certain level, after which it diminishes [25]. However, support for this hypothesis has been inconsistent [19]. The Ability Differentiation Hypothesis suggests that as intelligence increases, its correlation with specific abilities, like creativity, diminishes [26]. While many studies support this hypothesis, they often lack focus on the specific relationship between DT and Gf, using a wide range of ability tests instead [27].

Given the varied findings on the relationship between DT and Gf, accurately estimating their connection is challenging. It is crucial to recognize differences in definitions, measures, and study contexts [20]. These discrepancies highlight the need to consider broader factors, such as culture.

### Cultural comparisons in divergent thinking and fluid intelligence

Culture represents a group or nation’s shared beliefs, norms, customs, and behaviors [28,29]. These shared mental models influence thinking and provide culture-specific strategies for problem-solving [30].

A key cultural distinction is between collectivistic and individualistic societies [31]. Western cultures, seen as individualistic, emphasize personal qualities, achievement, and self-expression. In contrast, Eastern cultures prioritize community consensus, traditionalism, filial piety, and emotional self-control [32–35].

Creativity literature highlights the influence of cultural factors on perceptions and expressions of creativity [36–39]. Studies reveal cultural differences in how creativity is understood and manifested [40,41]. Key factors include varying definitions of creativity, distinct psychological processes, sociocultural environments that influence creative behavior, and the role of language [2]. Research generally shows that Westerners score higher than Easterners on creativity tests [40,41], though contrasting [42], neutral [43], and mixed [44] findings also exist.

Cultural influences on Gf show debated outcomes, with some studies indicating lower performance in Arab/Muslim samples [45,46]. However, research on deductive reasoning suggests that individuals from different cultures employ similar strategies [47].

### Current research

The present study explored cultural variations in the DT and Gf of the Russian and UAE samples. We aimed to investigate this relationship further by dissecting DT into its main components (fluency, originality, elaboration, and flexibility) to identify which components contribute most significantly to any emerging patterns in the relationship between DT and Gf. Our further goal was to investigate which ability holds a more substantial predictive influence on the other. Also, we intended to assess how cultural factors, specifically the country of origin, might impact the relationship between DT and Gf.

In recent years, numerous studies have emphasized the importance of cultural context in shaping both intelligence and creativity [48,49]. Variations in sociocultural environments, educational approaches, and cognitive frameworks were found to impact the development of these cognitive abilities significantly. The relevance of these studies to our research lies in the need for a cross-cultural comparison to understand better the interaction between DT and Gf across different cultural settings. By examining Russian and UAE samples, this study contributes to a growing body of literature that explores how cultural factors shape intellectual and creative capacities. Russia and the UAE present an intriguing comparison, as these countries exhibit contrasting educational systems, cultural values, and approaches to intellectual development. Literature suggests that societies with differing levels of individualism and collectivism, may foster distinct cognitive styles that affect both DT and Gf [50]. Furthermore, theoretical frameworks like Vygotsky’s sociocultural theory posit that cultural contexts shape the way cognitive processes, such as problem-solving and creativity, are developed [51]. Therefore, we hypothesize that these cultural differences will manifest in measurable variations in DT and Gf scores between the two groups.

We acknowledge that when conducting cross-cultural comparisons, particularly in cognitive domains like intelligence and creativity, it is essential to address the ethical considerations surrounding interpreting results. Comparisons of cognitive abilities across cultures can be misinterpreted as cultural superiority or inferiority measures. To mitigate these risks, we approach our findings through cultural relativism, acknowledging that each culture fosters unique cognitive strengths shaped by its specific sociocultural and educational environment [52]. Our analysis does not aim to rank cultures but rather to illuminate how different cultural factors influence the interplay between DT and Gf. Furthermore, we stress the need for caution in generalizing results, as our study is limited to two specific cultural groups. Indeed, our study has shed new light on these dynamics, revealing unique patterns that underscore the importance of cultural context in understanding the interplay between creativity and intelligence.

## Method

The American University of Sharjah Institutional Review Board approved the current study. All participants provided written consent to their participation.

### Participants

The sample comprised 106 participants, 53 from Russia (40 females) and 53 from the United Arab Emirates (UAE; 38 females). All study participants were undergraduate students from the most reputable universities in their respective countries: HSE University in Russia and the American University of Sharjah (AUS) in the UAE. These universities are comparable in their position in QS World University Rankings (www.topuniversities.com): In 2024, HSE was ranked 6th in Russia (399^th^ in the World Rating), and AUS was ranked 3rd in the UAE (364^th^ in the World Rating). Russian participants aged between 17 and 20 (*M *= 18.94, *SD *= 1.05), and UAE participants aged between 17 and 26 (*M *= 20.04, *SD *= 1.78). Participants received course credit for participation. Recruiting participants from prestigious universities may limit the generalizability of the findings to the broader population. However, our decision to sample from HSE University and the American University of Sharjah was made with the intent to control for educational quality and ensure that participants had comparable academic backgrounds across both cultural contexts. This approach allows us to focus on the cultural, rather than educational, differences in the relationship between DT and Gf. We employed a convenience sampling method, and, given the nature of this approach, a formal sample size calculation based on statistical power was not conducted.

### Procedure

Participants completed two tests: the Abbreviated Torrance Test for Adults (ATTA) as a measurement [53] of divergent thinking (DT) and the Culture Fair Intelligence Test (CFIT) as a measurement [54] of fluid intelligence (Gf).

The ATTA was selected primarily due to its efficiency in assessing DT across diverse groups. First, it is a quick-to-administer tool that captures multiple components of DT, including fluency, flexibility, originality, and elaboration. Second, ATTA was shown to have strong validity and reliability in various cultural settings, making it particularly useful in cross-cultural research. Third, its standardized scoring system allows for easier comparison between different populations, which is critical in cross-cultural studies like ours. Fourth, ATTA accesses DT across verbal and non-verbal tasks. This approach is critical in a cross-cultural study as it ensures that creativity is measured through multiple modalities, which helps mitigate cultural biases associated with language use and expression.

The Culture Fair Intelligence Test (CFIT) was selected due to its design to minimize cultural and linguistic biases in measuring fluid intelligence. First, it focuses on non-verbal reasoning tasks, which are less likely to be influenced by educational or language differences, making it suitable for cross-cultural comparisons. Second, CFIT was widely used in studies involving culturally diverse populations, and its robustness in reflecting cognitive ability without being swayed by participants’ cultural backgrounds enhances its validity in this type of research. Finally, its established use in global research enables more accurate benchmarking against findings from other studies on intelligence.

In cross-cultural studies, it is crucial to ensure that test translations maintain the original test’s integrity. UAE participants completed both tests in English, whereas Russian participants completed the tests in Russian. A back-translation procedure was used for both the ATTA and CFIT to ensure that the Russian versions of the tests preserved the original meaning and constructs as closely as possible [55]. Moreover, the tests were administered under similar conditions across both cultural groups to reduce any environmental or procedural biases. Age-related norms were considered.

### Instruments

#### Abbreviated torrance test for adults.

We used ATTA to assess the DT, which was developed based on the Torrance Tests of Creative Thinking [56] and consists of the same activities but in the abbreviated form, which requires less testing time. The ATTA was employed in a series of studies of Multilingual Creative Cognition [57] and demonstrated a good assessment of an individual’s DT. The ATTA manual reports the Kuder-Richardson (KR21) reliability coefficient for the total raw score for the four subscales (fluency, originality, elaboration, flexibility) as.84 [53].

The ATTA consisted of three paper and pencil activities, preceded by written instructions that explained general guidelines and encouraged participants to use their imagination and thinking abilities. In Activity 1, participants were asked to suppose that they could walk on air or fly and then identify the troubles they might encounter. In Activity 2, participants were presented with two incomplete figures and were asked to draw unusual pictures using these figures. In Activity 3, participants were presented with a group of nine triangles arranged in a 3x3 matrix and asked to draw as many pictures or objects as possible using those triangles.

Participants’ responses for three activities were assessed by four standard DT metrics, namely, fluency (quantity of ideas), originality (quality/uniqueness of ideas), elaboration (the number of details), and flexibility (ability to process information in different ways) by two independent raters fluent in English and Russian using standard ATTA assessment procedure [53]. Raw scores for the four metrics were obtained as a sum across initial raw scores for all/ chosen activities, as illustrated in Table 1. The significantly high inter-rater correlations between these raw scores by both raters (*r = *.82, *p* < .001) showed that ratings were comparable. Indexes produced by both raters were averaged to compute a creativity index (CI) score. The CI was used in subsequent analyses.

**Table 1:** ATTA activities across sub-scales.

№ Activity	Title	Mode	Fluency	Originality	Elaboration	Flexibility
1	Walk on air or fly	Verbal	x	x	–	–
2	Incomplete figures	Non-verbal	x	x	x	–
3	Triangles	Non-verbal	x	x	x	x

#### Culture fair intelligence test.

We used Scale 3 Form A from the Culture Fair Intelligence Test (CFIT) to assess [54] fluid intelligence (Gf). The CFIT manual reports the reliability coefficients for consistency over items as.74, test-retest consistency as.69, concept validity as.85, and concrete validity (calculated as a direct correlation with other intelligence tests) as.66.

The scale consists of four subtests (series, classification, matrices, conditions) containing different perceptual tasks. Before each subtest, the experimenter presented the instructions orally, and examples followed. In the Series subtest, participants were presented with an incomplete progressive series. Their task was to select а figure which best continues the series from the choices provided. In the Classification subtest, participants were exposed to five figures. They were asked to correctly identify two figures which were in some way different from the other three. In the Matrices subtest, the participants were asked to complete the matrix design presented at the left of each row. In the Conditions subset, participants were required to select from the five options the one that duplicated the conditions given in the far left box.

Following the recommended procedure, we summed the raw scores from all four subtests and transformed them into a normalized Gf score, taking participant age into account [54].

### Data analysis

At first, descriptive statistics were investigated. All scores were standardized into z-scores to screen the data for the univariate outliers. The threshold of 3.29 was used [58].

Relationships between CI and Gf scores were investigated using Pearson’s product-moment correlation coefficient for the total sample and the Russian and UAE samples separately (95% bootstrapped confidence intervals (CIs) based on 1000 samples were requested). In this study, we employed bootstrapped CIs to address potential limitations associated with our sample size. Bootstrapping is a powerful statistical technique that allows for robust estimation of CIs, mainly when dealing with small samples. Creating a large number of resamples of the observed dataset (in this case, 1000) offers an empirical approach to statistical inference and ensures more accurate and reliable confidence intervals for our correlation coefficients, enhancing our findings’ robustness. The resulting coefficients can be interpreted as effect size, where.10 indicates a small effect,.30 a medium effect, and.50 or above a large effect [59].

Six linear regression models were tested. In four of these models (Table 4: 1a and 1b, 3a and 3b), the Gf served as the dependent variable (DV), with the composite CI (Table 4: 1a and 1b) and the separate components of fluency, originality, elaboration, flexibility (Table 4: 3a and 3b) serving as independent variables (IV). The roles were reversed in the remaining two models (Table 4: 2a and 2b), with CI serving as the DV and Gf serving as the IV. Additionally, three models (Table 4: 1b, 2b, and 3b) included the country group as a factor, while the others (Table 4: 1a, 2a, and 3a) did not.

We employed segmented regression analysis to probe potential non-linear relationships among the CI and Gf to test the threshold hypothesis. At first, we fitted the model to the composite sample (*N* = 106). The present study automatically determined the breakpoint in our segmented regression analysis. Specifically, we utilized the ‘segmented()’ function from the segmented package in R. After the breakpoint detection, we divided the sample into two groups – below and above the breakpoint – and tested correlation coefficients in these groups. To ensure accuracy, we also conducted a sensitivity analysis when the scores equal to the breakpoint were moved from one group to another, and the correlations were re-run. A similar analysis was run separately for the country groups. However, due to even smaller sample sizes (*N* = 53 per group), the correlation coefficients were not estimated (as the frequency tables revealed, there were only nine participants above the breakpoint of 128 in the Russian sample and only four participants below the breakpoint of 100 in the UAE sample). Methodologically, our sample size was small to conduct such a complex analysis. Moreover, threshold hypotheses have faced criticism in recent research [19]. However, we still provide the results of this analysis and advise you to consider them cautiously.

A two-pronged approach was used to examine the differences in score distributions. It involved the analyses of variance ratios and the analysis of score distribution [60].

Levene’s test of Equality of Variances was used to examine differences in score variability, with a significant test indicating variance heterogeneity [17]. Variance ratios were calculated as measures of an effect size [60] by dividing the variance (*s²*) of the Russian sample by the variance (*s²*) of the UAE sample. Hence, values greater than 1.00 were considered a greater variability for the Russian sample than the UAE. A variance ratio greater than 1.10 or less than.90 suggests meaningful differences in variability. However, more conservative rules were also proposed [61]. The proportion ratio (*PR*) was calculated as the Russian sample size divided by the UAE sample size, equal to 1.00.

CI and Gf scores were standardized and transformed into z-scores to analyze the score distributions. The score distributions were divided into three regions based on the standard deviations from the mean: low scores (z < -1), scores around the mean (-1 < z < 1), and high scores (z > 1). Given that different patterns can emerge in these different regions, we can examine the tails and central region of the distribution separately. Upon segmenting the distribution, the proportions of each group in these regions were calculated and compared to assess the potential over-representation of certain groups in the different distribution regions. We ran chi-square (*χ2*) tests [17] to determine the statistical significance of the observed differences in the score distributions. Cramer’s *V* served as a measure of an effect size, with the coefficients of.1 for a small,.3 for a medium, and.5 for a large effect [59].

We conducted an Independent samples t-test to compare the CI and Gf scores between the Russian and UAE samples. Cohen’s *d* was used to measure effect size, with values of.2,.5, and.8 representing small, medium, and large effects, respectively [59]. To see the effect of the cultural group on the CI beyond the effect of Gf, ANCOVA was performed with the cultural group as an IV, the CI score as the DV, and the Gf as a covariate (CV). Four separate ANCOVAs were run further in which, instead of CI, four ATTA components, fluency, originality, flexibility, and elaboration, served as DV, with the cultural group as the IV and the Gf as a CV. Conversely, to see the effect of the cultural group on the Gf performance beyond the effect of CI, ANCOVA was performed with the cultural group as an IV, the Gf score as the DV, and the CI as a CV. Partial eta squared (*η²*) was utilized to measure effect size in ANCOVA tests. Values of.01,.06, and.14 represent small, medium, and large effect sizes, respectively [59].

In the current analysis, we do not present the results of the measurement invariance testing. Ensuring measurement invariance, crucial for meaningful cross-group comparisons, seeks to validate that the constructs measured are the same across different groups [62]. Therefore, establishing the measurement invariance is essential for comparing means of different groups [63]. The measurement invariance testing usually requires larger sample sizes [64] than is available in the current study. Although we proceed with cross-group comparisons, we acknowledge the caution that without establishing measurement invariance, the comparisons may not fully capture the intended constructs.

All analyses were conducted in JASP 0.18.1.0 and R 4.3.3.

## Results

Our study reveals several noteworthy patterns in the relationship between divergent thinking (DT) and fluid intelligence (Gf) across cultural contexts. First, although no significant correlations between creative index (CI) and Gf were found in either the Russian or the UAE samples, a significant negative relationship emerged when both samples were combined. Cross-cultural comparisons revealed significant differences in the distribution and variability of CI and Gf, highlighting distinct cognitive patterns across the two cultures. The Russian sample showed greater variability in creativity metrics, while the UAE sample exhibited more variation in fluid intelligence scores.

### Descriptive statistics

Descriptive statistics for the CI, fluency, originality, elaboration, flexibility, and Gf are available in Table 2. No missing data were found, and no univariate outliers were identified except for two cases (3.91 *SD* & 3.99 *SD*, equals 1.14%) in the Russian sample for elaboration. Due to the slight deviations from the suggested threshold (less than 1 *SD*), all cases (*N* = 106) were retained. Shapiro-Wilk’s test for the total sample returned significant results only for fluency and elaboration, with marginal significance for flexibility (*p* = .04). Measures of the central tendency, skewness, and kurtosis values indicated *p*otential deviations from the parametric assumptions for some scales, although they were not excessive.

**Table 2:** Descriptive Statistics.

Variable/ Statistic	Mean	Mode	Median	Min	Max	SD	Skewness	Kurtosis
**Country**	**Russia**							
**CI**	76.39	80.68	78	37	103	13.62	-.76	.88
**Gf**	112.94	114.86	113	76	145	13.81	-.47	.39
**Fluency**	16.19	16.48	16.5	11	19	2.48	-.8	-.29
**Originality**	16.3	16.43	16.5	11	19	1.94	-.6	.07
**Elaboration**	19.93	19.61	19.5	5	38	7.37	.42	.2
**Flexibility**	15.35	14.73	15.5	10	19	2.01	-.39	.24
**Country**	**UAE**							
**CI**	58.92	56.01	58	42	77	8.79	.1	-.4
**Gf**	133.85	144.14	137	78	178	21.02	-.48	-.12
**Fluency**	12.62	11.41	12	11	19	1.79	1.56	2.64
**Originality**	14.78	15.49	15	11.5	19	1.66	.01	-.35
**Elaboration**	12.85	12.87	12.5	5.5	23.5	4.21	.58	.22
**Flexibility**	12.98	13.07	13	10	18	1.7	.55	.76
**Country**	**Total**							
**CI**	67.65	59.71	66.75	37	103	14.40	.18	-.59
**Gf**	123.4	116.7	121	76	178	20.58	.14	-.29
**Fluency**	14.41	11.7^*^	14	11	19	2.8	.29	-1.34
**Originality**	15.54	15.92	15.5	11	19	1.95	-.13	-.54
**Elaboration**	16.39	13.5	15	5	38	6.95	.89	.79
**Flexibility**	14.17	14.11	14.5	10	19	2.2	.17	-.53

Note:

*More than one mode exists. CI - Creativity Index, Gf- Fluid Intelligence, UAE - United Arab Emirates, Min - Minimum Value, Max - Maximum Value, SD - Standard Deviation. Sample sizes were as follows: Russian: *N* = 53, UAE: *N* = 53.

### Divergent thinking and fluid intelligence link across countries

**Fig 1:**
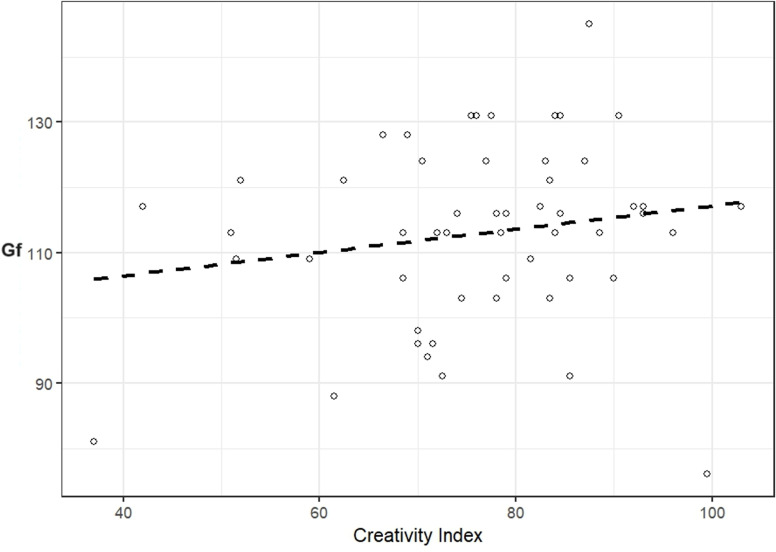
Scatterplot of the CI and Fluid Intelligence (Gf) relationship for the Russian sample.

**Fig 2:**
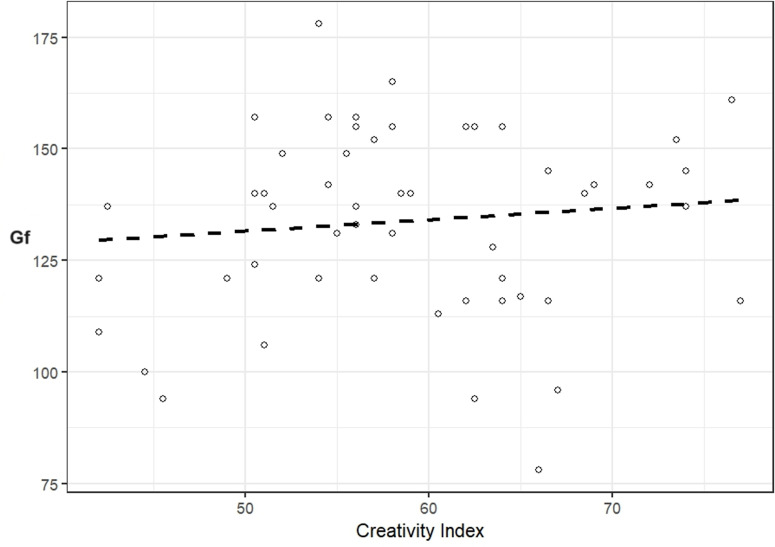
Scatterplot of the CI and Fluid Intelligence (Gf) relationship for the UAE sample.

Scatterplots of the CI-Gf relationship for the Russian and UAE samples are available in Figs 1 and 2, respectively. Scatterplots for Gf with fluency, originality, elaboration, and flexibility across Russian and UAE samples are available in the Supporting Information (S1 File, S1 and S2 Figs).

No significant relationships were found for the Russian (*r* = .18, 95% CIs [-.16;.46]) and UAE (*r* = .11, 95% CIs [-.16;.38]) samples. However, a significant negative correlation arose when the two samples were combined (*r* = -.22, 95% CIs [-.40; -.03], *p* = .02). Correlations between Gf and fluency, originality, elaboration, flexibility were non-significant for the Russian and UAE samples separately, but several correlations were significant for the total sample. Fluency, originality, elaboration, and flexibility significantly correlated across all samples, with effect sizes ranging from medium to large (see Table 3).

**Table 3:** Correlation analysis.

Variable	Country	Variable/ 95% Confidence Intervals [Lower bound, Upper bound]			
		Fluency	Originality	Elaboration	Flexibility
Gf	Russia	.22 [-.04;.48]	.22 [-.05;.50]	.07 [-.27;.41]	.17 [-.09;.45]
	UAE	-.09 [-.38;.19]	.15 [-.12;.42]	.09 [-.17;.35]	-.09 [-.35; 18]
	Total	-.29** [-.45; -.13]	-.06 [-.24;.11]	-.21* [-.39; -.02]	-.26** [-.41; -.09]
Fluency	Russia	—	.68*** [.48;.83]	.48*** [.24;.66]	.65*** [.46; 81]
	UAE	—	.56*** [.34;.73]	.38** [.20;.57]	.35* [.11;.62]
	Total	—	.70*** [.58;.78]	.62*** [.52;.71]	.69*** [.58;.79]
Originality	Russia	—	—	.32* [.00;.56]	.60*** [.30;.80]
	UAE	—	—	.53*** [.33;.69]	.30* [-.05;.55]
	Total	—	—	.50*** [.35;.64]	.58*** [.41;.72]
Elaboration	Russia	—	—	—	.36** [.08;.59]
	UAE	—	—	—	.55*** [.35;.74]
	Total	—	—	—	.58*** [.47;.68]

The results from testing six linear regression models are presented in Table 4. All models were statistically significant, with higher F ratios observed in the models that included the country factor. Models with fluency, originality, elaboration, and flexibility as separate independent variables explained more Gf variance than with CI as a single independent variable. The model that explained the largest amount of variance was Model 2b, in which Gf predicted CI, and the country factor was included.

The fitted segmented regression model showed that for each unit increase in Gf scores before reaching a breakpoint of 116 (95% CIs [95.61, 136.39]), CI increased by approximately.15 units. However, beyond this breakpoint, each additional unit increase in Gf was linked with a decrease of approximately.46 units in CI. The data were further divided into two parts: scores below the breakpoint (115 and below, *N* = 35: *N*_RUS_ = 27, *N*_UAE_ = 8) and above the breakpoint (116 and above, *N* = 71: *N*_RUS_ = 26, *N*_UAE_ = 45). The relationship between CI and Gf below the breakpoint was positive but non-significant (*r* = .09), while the relationship above the breakpoint was negative and significant (*r* = -.34, *p* = .003). Sensitivity analysis with nine participants with a score of 116 included in the ‘below the breakpoint’ group returned the same pattern. The breakpoint for the Russian sample alone was estimated to be 128 (95% CIs [100.42, 155.58]), and for the UAE sample, it was 100 (95% CIs [75.76, 124.24]). Correlations before and after the breakpoint were not estimated for the Russian and UAE samples due to underpower.

### Cross-cultural comparisons

**Fig 3:**
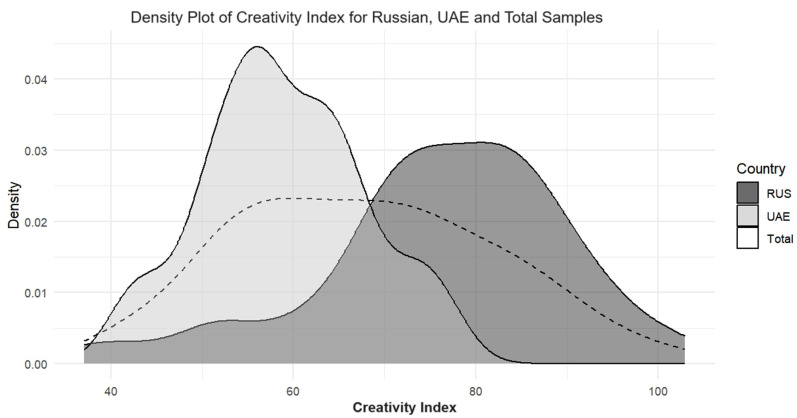
Creativity Index densities; RUS - Russian, UAE - United Arab Emirates.

**Fig 4:**
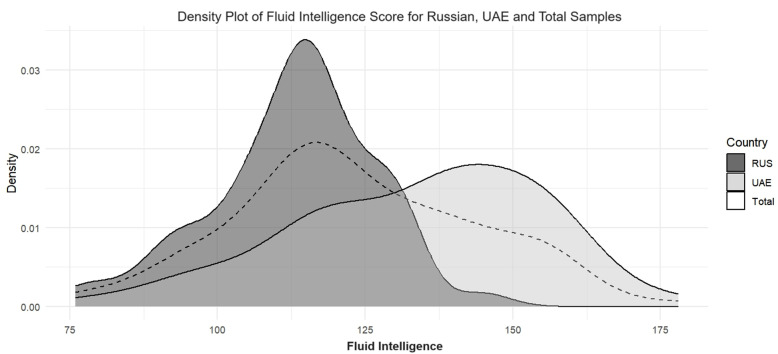
Fluid Intelligence densities: RUS - Russian, UAE - United Arab Emirates.

The CI and Gf densities across the Russian and UAE samples are available in Figs 3 and 4, respectively. Density plots for fluency, originality, elaboration, and flexibility can be found in the Supporting Information (S1 File, S3–S6 Figs). Figs 3 and 4 show minimal overlap between the score distributions among the samples. For the CI, scores are more spread out and flat in the Russian sample, while for the Gf, scores are more spread out and flat in the UAE sample.

The results from the variability analysis can be found in Table 5. Variance heterogeneity was only absent for the originality and flexibility sub-scales of the CI, as indicated by non-significant results from Levene’s test. In the CI, the Russian sample was more variable than the UAE sample, while in the Gf, the UAE sample was more variable. However, the highest variability was observed for the elaboration sub-scale in the Russian sample.

**Table 4:** Linear regression models.

Model	Variable	Status	R^2^	F	df	β u-st.	SE	β st.	t
1a	CI	IV	.05	5.42*	1, 104	-.32	.14	-.22	-2.239*
	Gf	DV				—	—	—	—
2a	CI	DV	.05	5.42*	1, 104	—	—	—	—
	Gf	IV				-.16	.07	-.22	-2.329*
3a	Fluency	IV	.14	4.02**	4, 101	-2.72	1.14	-.37	-2.39*
	Originality	IV				3.22	1.39	.31	2.32*
	Elaboration	IV				-.14	.36	-.05	-.38
	Flexibility	IV				-1.42	1.25	-.15	-1.13
	CFIT	DV				—	—	—	—
1b	CI	IV	.27	19.33***	2, 103	.20	.15	.14	1.33
	Country	Factor				24.43	4.34	—	5.63***
2b	GF	IV	.38	31.88***	2, 103	.08	.06	.12	1.329
	Country	Factor				-19.22	2.58	—	-7.45***
3b	Fluency	IV	.29	8.09***	5, 100	-.66	1.13	-.09	-.584
	Originality	IV				2.32	1.28	.22	1.807
	Elaboration	IV				.12	.34	.04	.346
	Flexibility	IV				-.57	1.16	-.06	-.496
	CFIT	Factor				21.53	4.68	—	4.599***

**Note.** ^ - DVs are omitted from the tables; however, they resemble the respective models without country factor: 1b - Gf, 2b - CI, 3b - Gf. CI - Creativity Index, Gf - Fluid Intelligence, IV - Independent Variable, DV - Dependent Variable, df- degrees of freedom, β u-st. - β unstandardized, SE - Standard Error, β st. - β standardised; *p < .05, **p < .01, ***p < .001.

**Table 5:** Variability Analysis.

Measure	Variance Ratios		Status	Levene’s Test	
	Calculation	Results		F	Sig
**CI**	185.54/ 77.34	2.4	RUS > UAE	5.4	.02
**Gf**	190.63/ 441.86	.43	UAE > RUS	11.11	<.001
**Fluency**	6.13/ 3.20	1.92	RUS > UAE	4.91	.03
**Originality**	3.75/ 2.75	1.36	RUS > UAE	.44	.51
**Elaboration**	54.32/ 17.70	3.07	RUS > UAE	10.59	<.001
**Flexibility**	4.03/ 2.90	1.39	RUS > UAE	2.19	.14

**Note:** CI - Creativity Index, Gf- Fluid Intelligence, RUS - Russian, UAE - United Arab Emirates. Proportion Ratios were the same for all variables: Russian/ UAE - 1.

The results from the distribution analysis can be found in Table 6. The chi-square (χ2) test results were statistically significant, indicating notable differences in CI and Gf level distributions between the Russian and UAE samples. The Cramer’s V values ranged from.36 to.60, suggesting that the strength of the association varied from medium to large [59]. For the CI in the UAE sample, most participants occupied the middle region of the distribution, whereas in the Russian sample, an equal number of participants were present in the middle and upper regions. Similar observations were made for fluency and flexibility. The opposite finding was observed for the Gf: in the Russian sample, almost all participants were in the middle region of the distribution, while in the UAE sample, an equally large percentage of participants were present in both the middle and upper regions. Moreover, the results for the originality and elaboration showed a comparable number of participants in the middle region in both the Russian and UAE samples.

**Table 6:** Distribution Analysis.

Variable	Region of the distribution	Russia		UAE		X^2^	Cramer’s V
		N	%	N	%		
**CI**	z < -1	2	3.77	9	16.98	73.02***	.57
	-1 < z < 1	22	41.51	42	79.25		
	z > 1	29	54.72	2	3.77		
**Gf**	z < -1	3	5.66	1	1.89	38.40***	.60
	-1 < z < 1	49	92.45	22	41.51		
	z > 1	1	1.89	30	56.6		
**Fluency**	z < -1	4	7.55	12	23.08	30.19***	.54
	-1 < z < 1	20	37.74	37	71.15		
	z > 1	29	54.72	3	5.77		
**Originality**	z < -1	4	7.55	11	20.75	13.76***	.36
	-1 < z < 1	32	60.38	39	73.58		
	z > 1	17	32.08	3	5.66		
**Elaboration**	z < -1	3	5.66	12	22.64	20.11***	.44
	-1 < z < 1	29	54.72	38	71.7		
	z > 1	21	39.62	3	5.66		
**Flexibility**	z < -1	3	5.66	9	16.98	24.65***	.49
	-1 < z < 1	23	43.4	40	75.47		
	z > 1	27	50.94	4	7.55		

**Note:** CI - Creativity Index, Gf – Fluid Intelligence, % is presented within the country.

Table 7 shows significant differences between the Russian and UAE samples across all research metrics, with the Russian sample scoring higher on the CI, fluency, originality, elaboration, and flexibility and the UAE sample scoring higher on the Gf.

**Table 7:** Independent Samples T-test and ANCOVA.

Metric	Statistic						
	Independent Samples T-test			ANCOVA			
	t	BF	d	F	F_CV_	n_p_^2^	n_p_^2^_CV_
**CI**	7.85***	Sig.	1.52	55.50***	1.77	.35	.02
**Gf**	-6.05***	Sig.	-1.18	31.64***	1.77	.24	.02
**Fluency**	8.5***	Sig.	.25	57.21***	.30	.36	0
**Originality**	4.33***	Non-Sig.	.21	21.76***	3.104	.17	.03
**Elaboration**	6.08***	Sig.	.23	31.04***	.49	.23	.01
**Flexibility**	6.55***	Non-Sig.	.23	32.80***	.06	.24	0

**Note.** ^ - The negative sign shows the UAE sample superiority. df for all were 104. CI - Creativity Index, Gf - Fluid Intelligence. BF - Brown-Forsythe test, if significant (p < .05), suggests violation of the equal variances assumption; *** p < .001, ** p < .01, * p < .05.

The cultural group significantly affected the CI when the effect of Gf was controlled for. However, the Gf as a covariate did not significantly affect the CI scores. The cultural group also significantly affected four ATTA components after controlling for the effect of Gf. Moreover, the cultural group significantly affected the Gf beyond the effect of CI.

## Discussion

### Relationship between divergent thinking and fluid intelligence

Investigating the relationship between divergent thinking (DT) and fluid intelligence (Gf) produced varied results across two cultural samples.

First, small correlations between the DT and Gf might reflect the actual size of the relationship [13,14]. The credibility of these modest correlations is underscored by their presence across both Russian and UAE samples despite marked disparities in the overall levels of the DT and Gf within each sample. This finding supports the divergent validity of the two constructs [12], bolstering research that considers DT and Gf as predominantly independent cognitive abilities [65–67]. However, we found that the 95% CIs were wide in a range between lower and upper bounds (app..5) and included zero for the CI and Gf across both samples. Such findings suggest that this might not represent accurate correlations, be artifacts, or suggest the presence of moderation effects [20].

It is plausible that a weak correlation between DT and Gf could be distorted due to a confluence of factors, such as cultural variations, measurement errors, or other underlying variables, and potential methodological or assessment artifacts such as task-, rater-, or procedure-specific factors [12]. For example, previous literature suggests correlations between DT and Gf depend on the measures used [13]. Moreover, while the previous studies have often focused on the relationship between observed DT and Gf, they have not consistently delved into latent variable models [16], which could potentially yield different correlation results. For example, an early study reported a correlation of *r* = .09 between intelligence and DT [14]. However, when the data was re-analyzed almost 50 years later, the strength of the relationship between latent factors of intelligence and DT increased (*r* = .20) once the actual variance was separated from the error variance [68]. Hence, it might be argued that the ‘true’ latent correlation between DT and Gf could be higher in our study, maintaining good discriminant validity, if other analytical strategies were implemented [17]. Although, they were impossible to perform due to the relatively small sample size, as stated in the limitations.

Furthermore, the relationship between DT and Gf significantly reversed, becoming negative when the total sample was investigated instead. This finding could be due to Simpson’s paradox [69], which occurs when a trend appears in different data groups but disappears or reverses when combined. This often occurs when a confounding variable is not accounted for in the analysis; for example, as in our study with the inclusion of the country group (suppressor), the regression coefficient for the CI reflected a direct effect on Gf within the same country group, which is positive, although non-significant [70]. In addition to Simpson’s paradox, the observed negative correlation when combining the samples could be explained by the variability within each sample. Notably, the Russian sample showed higher variability in CI, while the UAE sample demonstrated more variability in the CFIT. This finding can also be related to the unique composition of our sample, which comprises students from reputable universities, where the negative relationship might be sample-specific. Moreover, our findings hint at the complexity of the relationship between DT and Gf, which might not always be positive and could depend on various factors, including the characteristics of the sample and the measures used.

Moreover, when the country was not considered in the regression model, the CI explained approximately 5% of the variance in the Gf score, and vice versa, Gf explained approximately 5% in the CI score. However, when considered together, the country group and CI accounted for up to 27.3% of the variance in the Gf, and vice versa; the country group and the Gf explained around 38% of the variance in the CI, which is the largest amount explained among the research models in the current study. This finding indicates that the country group can play a significant role in predicting both Gf and DT. Our results might align with the Necessary But Not Sufficient Hypothesis, which suggests that high intelligence is a prerequisite for high creativity, potentially explaining why Gf with culture predicts DT variance more than the other way around [6]. This finding also aligns with the Ability Differentiation Hypothesis, which posits that the influence of Gf on DT might become less pronounced at higher intelligence levels [26]. Unfortunately, we could not test these theories explicitly due to the underpower of the current research. However, we tested one of the non-linear models, the Threshold Hypothesis [3]. In the total sample, the segment before the breakpoint demonstrated a non-significant positive trend, while the segment after the breakpoint demonstrated a significant negative trend.

To investigate the relationship between DT and Gf in greater depth, we further looked into the relationships between Gf and ATTA components: fluency, originality, elaboration, and flexibility, as we assumed that the composite CI score might have masked nuances of the DT and Gf relationship [12]. The correlations among the four components were from moderate to large in the total sample and country groups separately; together, they explained 13.7% of the variance in the Gf (compared to 5% of the variance explained by the composite CI). Furthermore, once the country group was added as a factor, the explained variance in the Gf increased to 29% (compared to 27.3% when the composite CI was used). For the total sample, fluency, elaboration, and flexibility supported the overall negative association with the Gf; originality - resulted in the non-significant, nearly zero relationship with the Gf. However, the 95% CIs for the coefficients were wide and included zero, indicating a potential lack of robustness. Previous research suggested that originality might function independently of other creativity components and involve unique processes that may not correlate with more generalized cognitive abilities [12,19,68]. Moreover, research in the field of Multilingual Creative Cognition [71] suggests that creativity components can be categorized into two types of creative functioning: fluency, flexibility, and elaboration to be associated with a ‘generative capacity,’ the ability to activate and work through a multitude of unrelated concepts; originality to be associated with an ‘innovative capacity,’ the ability to produce original and valuable ideas. Although, once holding other components constant, only two predictors were significant: as fluency scores increased, the Gf decreased, and conversely, an increase in originality corresponded with an increase in the Gf.

### Cultural comparisons in divergent thinking and fluid intelligence

Our study demonstrated potential cultural differences in the performance of DT and Gf tests. The Russian sample had higher average scores and greater score diversity in the CI, while the UAE sample had higher average scores and displayed more variability in the Gf scores. The higher DT performance of the Russian sample compared to the UAE sample aligns with research highlighting cross-cultural differences in the DT, specifically, the notion that Eastern countries/ collectivist environments might limit creativity [71]. However, West versus East categorization might be too simplistic, and attributing each study country to either side might not be accurate. Although Russia scored lower on individualism than, for example, the US [32], it underwent some shift towards a less collectivist society. While the UAE rapidly changes towards Westernization, it retains a solid collectivistic and Islamic identity.

Moreover, while Western participants typically show an advantage on DT tasks [71], it is essential to consider that most creativity tests are grounded in Western frameworks [6]. Research suggests these tests might miss aspects of creativity valued in Eastern cultures [72], and they may assess Easterners’ familiarity with Western thinking styles rather than their true creative potential [73]. However, the finding that the UAE participants outscored the Russian ones on the Gf might involve methodological underpinnings. Although the CFIT employed in the present study claims to be culture fair, it may be sensitive to certain aspects of Gf that are prevalent in the Emirati (more Eastern) culture but not in Russian (more Western) culture. This consideration brings us to the cultural specificity issue in the implicit intelligence theory and the validity of the CFIT [74].

The UAE sample’s CI scores were more concentrated in the middle region of the distribution, with few scores at high or low ends. The Russian sample’s CI scores were spread across the middle and upper regions. The same but reversed patterns were observed for the Gf: the Russian sample’s Gf scores were primarily concentrated in the middle region, with fewer participants at low or high ends; the UAE sample’s Gf scores spanned the distribution’s middle and upper regions. Concentrated scores in the UAE sample for CI and the Russian sample for Gf suggest a more uniform level of DT or Gf, respectively. However, even though the Russian sample outperformed the UAE sample across all the DT components, effect sizes and density overlaps varied. The most pronounced difference was observed in fluency, with the least overlap in densities, suggesting that fluency might be a critical differentiator between the two samples. Contrastingly, originality yielded the slightest difference between the two samples, with the most significant density overlap and homogeneity of variances, indicating similar originality levels across both countries. Different cognitive strategies influenced by Gf could potentially explain these differences in fluency, as individuals with higher Gf may not exhibit the serial-order effect. Instead, higher Gf may enable the rapid direction of attention toward less conventional ideas from the onset of creative thinking, which could result in fewer but more original responses [75,76].

It might be the case that the measurements of Gf and DT may not be directly comparable due to their inherent nature [12]. Gf measures a more concrete ability, as it is challenging to manipulate intellectual capacity. On the other hand, DT might be more susceptible to influence from various factors such as personal choice, motivation, and cultural context and possess more degrees of freedom. Therefore, creativity is often viewed as a product of a system of characteristics rather than a single, isolatable trait [12]. As suggested earlier, many individuals possess the capacity to be creative but choose not to utilize it [77]. For example, the cultural environment of the UAE appears to be more restrictive toward creative expression, which might influence the allocation of cognitive and personal resources of the UAE sample towards intellectual activities (Gf) rather than creative activities (DT).

Moreover, factors such as intrinsic motivation and perceived fairness of the work environment, which were not considered in the current study, can influence the DT output [12]. High motivation alone may not guarantee high performance, especially when cognitive ability or task-related knowledge is lacking. However, cognitive ability or task-related knowledge does not ensure the best results in performance if there is no motivation to do so [78].

### Practical implications

The findings of this study underscore the importance of considering cultural contexts when applying divergent thinking (DT) and fluid intelligence (Gf) assessments in educational and psychological practices. Understanding the distinct cognitive profiles of individuals from different cultural backgrounds can inform tailored interventions and instructional strategies that leverage their unique strengths. For instance, educators and psychologists should recognize that the methodologies used in creativity assessments may favor specific cultural paradigms, potentially disadvantaging individuals from non-Western backgrounds. It is crucial to incorporate diverse creative assessment tools that reflect cultural values and learning styles to enhance educational practices. Furthermore, given the complexities identified in the relationship between DT and Gf, practitioners should adopt a holistic approach that integrates both constructs. Training programs could be developed that enhance cognitive abilities and stimulate creative potential, particularly in cultures where creative expression may be less emphasized.

## Limitations and further research

Our study has several limitations. First, our reliance on correlational analyses restricted causal inferences, while the relatively small sample size prevented us from directly testing complex latent models or non-linear patterns (e.g., Necessary But Not Sufficient Hypothesis, Ability Differentiation Hypothesis). Even though we tested the Threshold Hypothesis, the interpretation of the findings is complex. The ideal approach would involve using Gf as a continuous moderator rather than searching for a single breakpoint [17,19,27]. Finally, we only considered the country as a potential moderator, leaving a significant portion of the unexplained variance beyond our consideration. Future cross-cultural research on the dynamics between the DT and Gf with larger samples could delve deeper into these complexities by employing more sophisticated analyses and considering a wider range of contextual factors [12].

## Conclusions

We investigated the relationship between DT and Gf in two culturally different samples. While our results reveal differences between the Russian and UAE samples, we suggest not viewing them as a straightforward comparison of cultural differences. Instead, they offer a deeper understanding of the complex interplay between cognitive ability and creativity.

## Supporting information

S1 File Supplementary scatterplots and density distributions illustrating associations between fluid intelligence (Gf), divergent thinking measures (fluency, originality, elaboration, flexibility), and cross-national comparisons across Russian and UAE samples. (DOC)

S2 File Includes research data table used for the data analysis. (DOC)
