# Supplementary material for: Unravelling the tapestry: Cross-cultural insights into intelligence and creativity
Source: PLoS One. 2025 May 6;20(5):e0320942. doi: 10.1371/journal.pone.0320942 (PMC12054861; doi:10.1371/journal.pone.0320942)

**Supporting Information**

**S1 Figure** - Scatterplots for Gf with Fluency, Originality, Elaboration, Flexibility for the Russian Sample


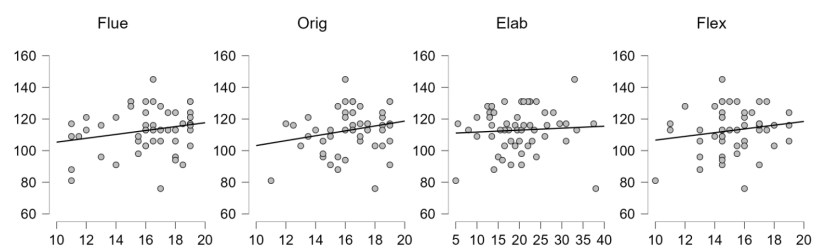


Y axis: Fluid Intelligence

X axis: Flue - Fluency, Orig - Originality, Elab - Elaboration, Flex - Flexibility

**S2 Figure** - Scatterplots for Gf with Fluency, Originality, Elaboration, Flexibility for the United Arab Emirates Sample


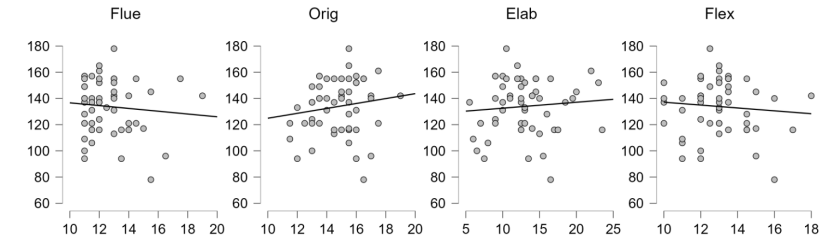


Y axis: Fluid Intelligence

X axis: Flue - Fluency, Orig - Originality, Elab - Elaboration, Flex - Flexibility

**S3 Figure** - Densities for Fluency across Russian (RUS) and United Arab Emirates (UAE) samples


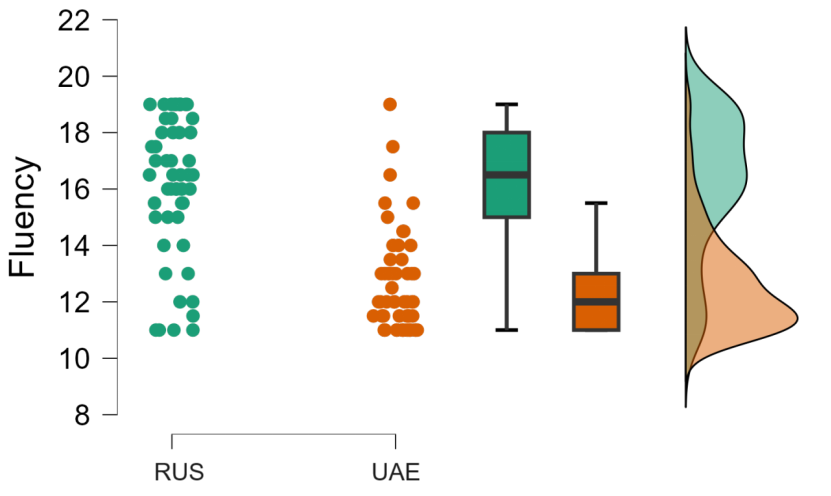


**S4 Figure** - Densities for Originality across Russian (RUS) and United Arab Emirates (UAE) samples


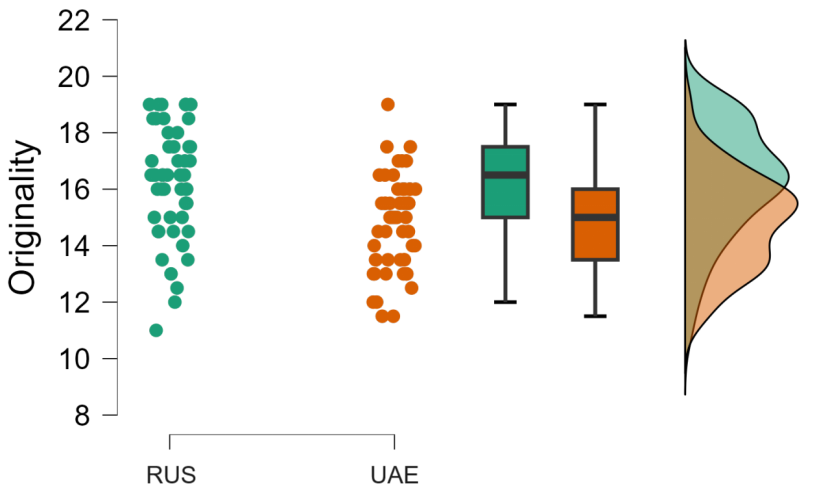


**S5 Figure** - Densities for Elaboration across Russian (RUS) and United Arab Emirates (UAE) samples


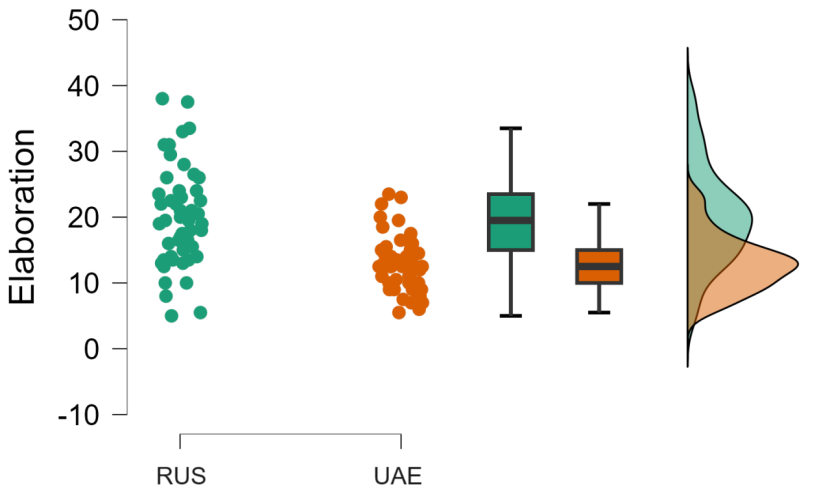


**S6 Figure** - Densities for Flexibility across Russian (RUS) and United Arab Emirates (UAE) samples


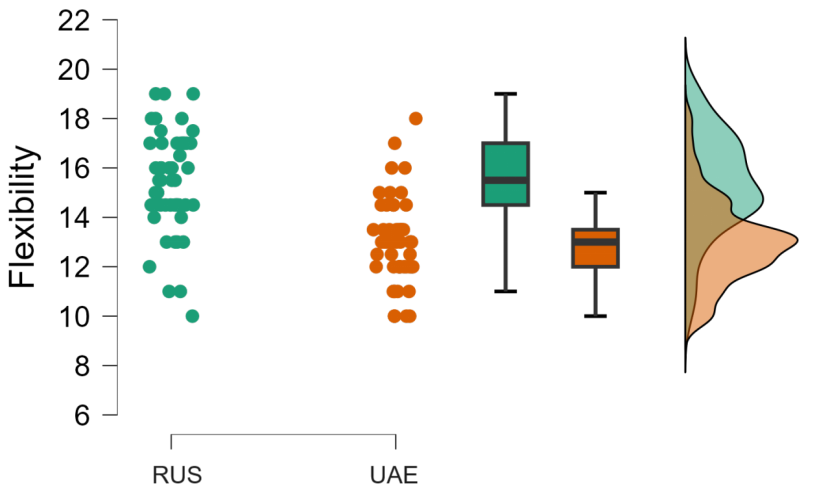

Supplement: S1 File — (DOC) [file pone.0320942.s001.doc]
