# Supplementary material for: Unravelling the tapestry: Cross-cultural insights into intelligence and creativity
Source: PLoS One. 2025 May 6;20(5):e0320942. doi: 10.1371/journal.pone.0320942 (PMC12054861; doi:10.1371/journal.pone.0320942)
Supplement: S2 File — (DOC) [file pone.0320942.s002.doc]

**Research Data**

| Participant | Country | Creativity Index | CFIT | Fluency | Originality | Elaboration | Flexibility |
| --- | --- | --- | --- | --- | --- | --- | --- |
| 7201 | RUS | 68.5 | 106 | 17 | 14.5 | 20 | 13 |
| 7202 | RUS | 83.5 | 103 | 19 | 18.5 | 19 | 18 |
| 7203 | RUS | 42 | 117 | 11 | 12 | 5.5 | 11 |
| 7204 | RUS | 84 | 113 | 18 | 17.5 | 23.5 | 13 |
| 7205 | RUS | 93 | 116 | 19 | 18.5 | 26.5 | 18 |
| 7206 | RUS | 84.5 | 131 | 19 | 19 | 16.5 | 17.5 |
| 7207 | RUS | 82.5 | 117 | 19 | 17 | 17 | 17 |
| 7208 | RUS | 37 | 81 | 11 | 11 | 5 | 10 |
| 7209 | RUS | 79 | 106 | 16.5 | 17 | 18 | 19 |
| 7210 | RUS | 90.5 | 131 | 19 | 19 | 20.5 | 17 |
| 7211 | RUS | 87 | 124 | 19 | 16.5 | 26 | 14.5 |
| 7212 | RUS | 83.5 | 121 | 19 | 18.5 | 19.5 | 16 |
| 7213 | RUS | 74.5 | 103 | 16 | 18 | 16.5 | 15.5 |
| 7214 | RUS | 74 | 116 | 13 | 16.5 | 19 | 16 |
| 7215 | RUS | 90 | 106 | 18 | 19 | 23 | 16 |
| 7216 | RUS | 88.5 | 113 | 19 | 18.5 | 19.5 | 17.5 |
| 7217 | RUS | 85.5 | 91 | 18.5 | 19 | 18 | 17 |
| 7218 | RUS | 84 | 131 | 15 | 16.5 | 22.5 | 15 |
| 7219 | RUS | 78.5 | 113 | 16 | 16.5 | 20.5 | 15.5 |
| 7220 | RUS | 61.5 | 88 | 11 | 15.5 | 14 | 13 |
| 7221 | RUS | 71.5 | 96 | 13 | 14.5 | 24 | 14.5 |
| 7222 | RUS | 75.5 | 131 | 16 | 16 | 24 | 15.5 |
| 7223 | RUS | 72 | 113 | 17.5 | 15 | 17.5 | 17 |
| 7224 | RUS | 103 | 117 | 18.5 | 19 | 37.5 | 17 |
| 7225 | RUS | 78 | 116 | 16.5 | 12.5 | 22.5 | 18 |
| 7226 | RUS | 83 | 124 | 17.5 | 16 | 28 | 16.5 |
| 7227 | RUS | 79 | 116 | 16 | 16.5 | 21 | 17 |
| 7228 | RUS | 70.5 | 124 | 16 | 16.5 | 13 | 16 |
| 7229 | RUS | 70 | 96 | 18 | 15.5 | 15 | 13 |
| 7230 | RUS | 99.5 | 76 | 17 | 18 | 38 | 16 |
| 7231 | RUS | 73 | 113 | 16.5 | 17.5 | 16.5 | 14.5 |
| 7232 | RUS | 78 | 103 | 16 | 13 | 22 | 16 |
| 7233 | RUS | 85.5 | 106 | 15.5 | 14.5 | 31 | 14.5 |
| 7234 | RUS | 69 | 128 | 17 | 15.5 | 12.5 | 14 |
| 7235 | RUS | 87.5 | 145 | 16.5 | 16 | 33 | 14.5 |
| 7236 | RUS | 72.5 | 91 | 14 | 15 | 20.5 | 14.5 |
| 7237 | RUS | 77.5 | 131 | 15 | 16.5 | 22 | 14.5 |
| 7238 | RUS | 66.5 | 128 | 15 | 17 | 13.5 | 12 |
| 7239 | RUS | 96 | 113 | 17 | 16 | 33.5 | 15 |
| 7242 | RUS | 92 | 117 | 18.5 | 17.5 | 29.5 | 17 |
| 7243 | RUS | 93 | 117 | 19 | 17.5 | 31 | 17 |
| 7244 | RUS | 52 | 121 | 12 | 13.5 | 10 | 14.5 |
| 7245 | RUS | 81.5 | 109 | 15.5 | 15 | 26 | 14.5 |
| 7246 | RUS | 51 | 113 | 12 | 14 | 8 | 11 |
| 7247 | RUS | 62.5 | 121 | 14 | 16 | 13 | 14.5 |
| 7248 | RUS | 59 | 109 | 11 | 17 | 13.5 | 14 |
| 7249 | RUS | 71 | 94 | 18 | 16 | 16 | 14.5 |
| 7250 | RUS | 51.5 | 109 | 11.5 | 13.5 | 10 | 14.5 |
| 7251 | RUS | 70 | 98 | 15.5 | 14.5 | 20.5 | 15.5 |
| 7252 | RUS | 68.5 | 113 | 16 | 16.5 | 15.5 | 14.5 |
| 7253 | RUS | 76 | 131 | 16.5 | 16.5 | 21 | 14.5 |
| 7255 | RUS | 77 | 124 | 18 | 18.5 | 14 | 19 |
| 7256 | RUS | 84.5 | 116 | 19 | 19 | 13.5 | 19 |
| 40624 | UAE | 54.5 | 142 | 13 | 15 | 11 | 12 |
| 40636 | UAE | 74 | 137 | 12 | 15.5 | 18.5 | 13.5 |
| 40645 | UAE | 54.5 | 157 | 11 | 13.5 | 10 | 12 |
| 40654 | UAE | 62.5 | 94 | 13.5 | 16 | 13.5 | 11 |
| 40657 | UAE | 76.5 | 161 | 12 | 17.5 | 22 | 13 |
| 40681 | UAE | 66 | 78 | 15.5 | 16.5 | 16.5 | 16 |
| 40687 | UAE | 50.5 | 124 | 12 | 13 | 9 | 12 |
| 40708 | UAE | 58 | 165 | 12 | 15.5 | 12 | 13 |
| 40711 | UAE | 74 | 145 | 15.5 | 15 | 20 | 15 |
| 40717 | UAE | 62.5 | 155 | 13 | 16.5 | 12.5 | 14.5 |
| 40723 | UAE | 50.5 | 140 | 11 | 13.5 | 11 | 11 |
| 40759 | UAE | 57 | 152 | 12 | 16 | 13 | 10 |
| 40765 | UAE | 45.5 | 94 | 11 | 12 | 7.5 | 12 |
| 40771 | UAE | 60.5 | 113 | 13 | 14.5 | 15 | 13 |
| 40777 | UAE | 54 | 121 | 11.5 | 12.5 | 9 | 14.5 |
| 40780 | UAE | 72 | 142 | 19 | 19 | 14 | 12 |
| 40783 | UAE | 56 | 137 | 11 | 14.5 | 12.5 | 12 |
| 40792 | UAE | 58 | 131 | 13 | 14 | 13 | 11 |
| 40804 | UAE | 62.5 | 155 | 17.5 | 15.5 | 12 | 12 |
| 40819 | UAE | 66.5 | 116 | 11.5 | 15.5 | 17 | 13.5 |
| 40828 | UAE | 63.5 | 128 | 11 | 15.5 | 16 | 14.5 |
| 40849 | UAE | 42 | 121 | 11 | 11.5 | 7 | 10 |
| 40855 | UAE | 55 | 131 | 11.5 | 16 | 10 | 13 |
| 40873 | UAE | 50.5 | 157 | 11.5 | 13.5 | 9 | 12 |
| 40885 | UAE | 56 | 133 | 12.5 | 12 | 13 | 13 |
| 40894 | UAE | 51 | 106 | 11.5 | 15.5 | 8 | 11 |
| 40936 | UAE | 65 | 117 | 15 | 15.5 | 14 | 15 |
| 40966 | UAE | 56 | 157 | 11 | 16 | 9 | 13.5 |
| 40975 | UAE | 56 | 155 | 13 | 14.5 | 10.5 | 13 |
| 40987 | UAE | 51 | 140 | 12 | 14 | 9 | 12 |
| 40990 | UAE | 54 | 178 | 13 | 15.5 | 10.5 | 12.5 |
| 40996 | UAE | 66.5 | 145 | 13 | 15.5 | 14.5 | 13.5 |
| 41038 | UAE | 67 | 96 | 16.5 | 17 | 15.5 | 15 |
| 41044 | UAE | 51.5 | 137 | 12 | 13 | 10 | 13 |
| 41053 | UAE | 62 | 116 | 13.5 | 16 | 12.5 | 14.5 |
| 41059 | UAE | 64 | 121 | 14.5 | 17.5 | 12.5 | 13 |
| 41089 | UAE | 42 | 109 | 11 | 11.5 | 6 | 11 |
| 41095 | UAE | 62 | 155 | 11 | 14 | 14.5 | 13.5 |
| 41098 | UAE | 69 | 142 | 14 | 17 | 14 | 18 |
| 41101 | UAE | 55.5 | 149 | 11 | 13.5 | 12.5 | 13.5 |
| 41122 | UAE | 73.5 | 152 | 13 | 16 | 23 | 13 |
| 41128 | UAE | 58.5 | 140 | 11.5 | 13.5 | 12.5 | 13 |
| 41155 | UAE | 64 | 155 | 14.5 | 16.5 | 16.5 | 13.5 |
| 41167 | UAE | 49 | 121 | 11 | 13.5 | 7 | 12.5 |
| 41179 | UAE | 44.5 | 100 | 11 | 13 | 6.5 | 12 |
| 41194 | UAE | 68.5 | 140 | 13 | 15 | 19.5 | 16 |
| 41203 | UAE | 52 | 149 | 11 | 13 | 10 | 12.5 |
| 41230 | UAE | 42.5 | 137 | 11.5 | 13 | 5.5 | 10 |
| 41239 | UAE | 59 | 140 | 11 | 17 | 15 | 10 |
| 41257 | UAE | 64 | 116 | 12 | 15 | 17.5 | 14.5 |
| 41281 | UAE | 58 | 155 | 12 | 15 | 14.5 | 13.5 |
| 41374 | UAE | 77 | 116 | 14 | 14.5 | 23.5 | 17 |
| 41467 | UAE | 57 | 121 | 14 | 13 | 13 | 13 |
